# Partner Notification: A Community Viewpoint

**DOI:** 10.1002/jia2.25291

**Published:** 2019-07-19

**Authors:** George Ayala, Mahri Bahati, Elie Balan, Judy Chang, Tri D Do, Najeeb A Fokeerbux, Abdulwahid Hassan, Julien Kerboghossian, Midnight Poonkasetwatana, Jorge Saavedra, Andrew Spieldenner, Ruth M Thomas, Johnny Tohme, Jeffrey Walimba

**Affiliations:** ^1^ MPact Global Action for Gay Men's Health and Rights Oakland CA USA; ^2^ University of California San Francisco CA USA; ^3^ Innovative Response Globally to Transgender Women and HIV (IRGT) San Francisco CA USA; ^4^ M‐Coalition Beirut Lebanon; ^5^ International Network of People Who Use Drugs (INPUD) London United Kingdom; ^6^ Young Queer Alliance Port Louis Mauritius; ^7^ Amkeni Malinda Nairobi Kenya; ^8^ Julien Kerboghossian Global Network of People Living with HIV (GNP+) Amsterdam NL; ^9^ APCOM Bangkok Thailand; ^10^ AIDS Healthcare Foundation Miami FL USA; ^11^ California State University San Marcos CA USA; ^12^ U.S. People Living with HIV Caucus San Diego California USA; ^13^ Global Network of Sex Work Projects Edinburgh Scotland; ^14^ Ishtar MSM Nairobi Kenya

**Keywords:** HIV, partner notification, community, key populations, people living with HIV

Partner notification is a voluntary process, in which trained health workers ask people with HIV about their sexual and/or injecting partners and then, with consent, contact these partners to offer HIV testing services. Partner notification can be: passive (an individual diagnosed with HIV is encouraged by a health worker to disclose, on their own, their status to sexual and injecting partners, then suggest their partner(s) receive an HIV test); or assisted (person diagnosed with HIV consents to having a trained provider disclose on their behalf and then offer HIV testing to their partner(s)). Many individuals prefer the option of provider‐assisted service referrals for sexual or drug using partners without disclosure. This definition is internationally recognized and included in the World Health Organization (WHO) published global guidelines. The WHO guidelines describe the rights‐based principles of consent, confidentiality, counselling, correct test results, and connection to services for all HIV testing and partner notification services 1.

While partner notification is a welcome addition to the public health toolbox of interventions designed to address HIV, it is viewed with cautious apprehension by community advocates and healthcare recipients. And for good reason: violations of privacy, breaches in confidentiality and coercive medical practices are commonly experienced by immigrants, lesbian, gay, bisexual, transgender and intersex people, people of colour, people who use drugs, sex workers and women worldwide 2, 3, 4. The consequences of such violations can be particularly dire for these groups. In many countries, one or more of these groups are criminalized and/or at increased risk for violence 5, enacted with impunity by neighbours, co‐workers, healthcare providers, police and other State actors 6.

This community viewpoint aims to highlight partner notification services implemented in ways that are perceived as unethically misaligned with global guidance, using the subjective experiences of people living with and affected by HIV. This viewpoint is not a response to the papers included in this issue and therefore does not reflect on research findings or practices described herein. Instead, our paper offers a set of general recommendations to minimize harm and to optimize the benefits of partner notification services, with an emphasis on considerations needed when engaging socially marginalized and/or criminalized populations.

## Harmful public health practices

Abandoning informed consent, violating privacy and confidentiality, and failing to connect individuals to quality, evidence‐based services they need are examples of harmful public health practices commonly experienced by socially marginalized and/or criminalized groups 7, 8. Global and national donors to local public health programmes, including HIV testing services, may be unknowingly contributing to such practices by over‐emphasizing “case‐finding” and “yield” in the expectations they set for grantees 9, 10. Under these circumstances, there is a real risk of rapid scale‐up of partner notification services without proper ethical procedures in place.

In some countries and with certain populations, like gay men, migrants, people who inject drugs, sex workers and transgender people, HIV testing and partner notification services, are mandatory 11, 12, and are seriously deviating from the standards set by the WHO. In some places, governments are given augmented authority to identify and contact the sex or injecting partners of people with HIV without consent. In other instances, healthcare providers may simply be perceived by the public to have such authority 13.

In countries that criminalize same‐sex sexual behaviour, drug use, gender non‐binary expression or behaviour, and sex work, authorities may conduct random arrests and forced HIV testing 14, 15. Moreover, HIV testing and partner notification without consent or reliable assurances of confidentiality is unethical and creates powerful social disincentives for seeking services. Legal provisions to protect HIV‐positive individuals against potential harm following unconsented disclosure from substandard partner notification services may be lacking 16, 17. Stigma associated with diagnosed HIV and public disclosure about one's sexual and/or drug using practices have resulted in loss of jobs, health insurance, homes, social connection and support services 18.

Despite WHO guidelines 1, HIV testing and partner notifications services are not always offered holistically, in combination with, or integrated as part of more comprehensive health programmes. As a result, a positive HIV test result may not necessarily lead to immediate access to care and treatment 19, undermining the public health potential of partner notification services. Although linkage to treatment after a positive HIV test is becoming the norm, stigma and discrimination still complicate efforts to ensure a seamless and immediate linkage to care. In addition, renewed efforts to encourage the uptake of normative guidance are still needed, especially given that 40% of people living with HIV (PLHIV) (of which 25% remain unaware of their status), are without treatment 20, 21. Additionally, counselling services, including counselling to assess, prevent or mitigate violence (i.e. intimate partner violence, violence associated with being a member of a key population or living with HIV), is under‐funded or seldomly offered as part of a complete package of services. This is a missed opportunity because partner notification, when linked with other services, can increase access and uptake of HIV testing, prevention, care and treatment 22, 23. Linkages to and from other supportive services like mental health, substance abuse treatment, and violence prevention and treatment, would be ideal; however, these services are not always made available.

## Other challenges to good practice

Although reports of harm resulting from partner notification in research studies are rare 1, 24, harm may go unreported in places where stigma, discrimination, violence, blackmail, extortion and arrests go unchecked 25. Often this is because there are no safe or clearly articulated mechanisms available for reporting the risk for or experiences of violence. Hence, the promise of anonymity is central to many successful HIV testing programmes 26. Partner notification, when not implemented in line with the evidence‐informed and rights‐based WHO guidelines, could threaten the success of those programmes, given the risk of people not showing up for testing out of fear that anonymity cannot be guaranteed.

Having multiple concurrent sexual and injecting partners also complicates partner notification and may add to existing stigma experienced by PLHIV. Sexual and injecting partners may not be open to being contacted, and partners may feel “outed.” Involuntary or unintentional disclosure about the sexual orientation, sexual identities, gender identities, and sexual and injecting practices of partners may be unexpected, unwelcome and perceived as an egregious violation of one's privacy, especially to men who have sex with men, people who inject drugs, sex workers and their clients, and transgender people. The providers of partner notification services must be well‐trained and sensitized to the unique needs of key populations and other socially marginalized groups. Training and sensitization can help providers to more carefully tailor services when working with key populations.

Finally, many developed countries routinely conduct HIV drug resistance testing to select appropriate treatment regimens. This technology can be used to locate transmission clusters (networks) that may otherwise go unrecognized 27. Although not included in partner notification approaches, there are community fears that molecular HIV surveillance can be used to identify undocumented immigrants and criminalized populations living with HIV. Misinformation and fear have a chilling effect, lowering the chances that people will ask for or consent to partner notification. This underscores the importance of carefully crafted and targeted educational efforts about the public health benefits of partner notification and other HIV services.

## Recommendations

Trust is key to partner notification services and earned when community members feel that their privacy, consent and confidentiality, and that of their network members, are protected. The following are a few good practice recommendations for inspiring community trust:

1. Offer voluntary partner notification services but only after informed consent is given. Pay special attention to individuals under the age of consent and others who cannot legally give consent.
2. Do not offer partner notification services where PLHIV and other socially marginalized groups are criminalized, if the risks of doing so outweigh the benefits.
3. Engage communities in the design, implementation and evaluation of partner notification services, especially PLHIV, marginalized and/or criminalized populations.
4. Fully fund community‐led organizations and peer‐led programmes to deliver technically competent, high‐quality voluntary partner notification services, with an emphasis on the potential benefits, including safe disclosure over time led by PLHIV, extension of pre‐exposure prophylaxis (PrEP) for heterosexual and same‐sex sero‐discordant couples, and the use of anonymous approaches.
5. Train and sensitize healthcare providers to deliver rights‐based, partner notification services, with consideration for the specific needs and concerns of immigrants, lesbian, gay, bisexual, transgender and intersex people, people of colour, people who use drugs, sex workers and women.
6. Accurately and comprehensively assess the risk for violence (intimate partner and family violence, gender‐based violence, and violence associated with drug use, gender identity, sexual orientation, sex work, poverty and xenophobia) before offering voluntary partner notification services.
7. Ensure and offer counselling services, including counselling to prevent or mitigate violence.
8. Disseminate fact‐based information about voluntary partner notification programmes in partnership with communities and in tandem with community‐led “know‐your‐rights” campaigns.

Community advocates and healthcare recipients, including PLHIV, can be strong contributors to the implementation of rights‐based public health programmes, including partner notification services 28, 29. We have an obligation to ensure the highest attainable standard of physical and mental health, which includes unfettered access to services 30. This includes upholding the principles of self‐determination and bodily integrity 31, which should be prioritized above all else when implementing partner notification services.

## Competing interests

Authors have no competing interests to declare.

## Authors’ contributions

GA is the lead writer, facilitated a series of online consultations and synthesized themes related to partner notification. All co‐authors participated equally in online consultations, sharing community reports and inputting into early drafts of the manuscript.
